# Supplementary material for: Skeletal muscle-secreted DLPC orchestrates systemic energy homeostasis by enhancing adipose browning
Source: Nat Commun. 2023 Nov 30;14:7916. doi: 10.1038/s41467-023-43402-z (PMC10689447; doi:10.1038/s41467-023-43402-z)
Supplement: Supplementary file 1 — Supplementary Information [file 41467_2023_43402_MOESM1_ESM.pdf]

Supplementary information for:

Skeletal muscle-secreted DLPC orchestrates systemic energy homeostasis by enhancing adipose browning

Xiaodi Hu[1,#], Mingwei Sun[2,#], Qian Chen[1], Yixia Zhao[1], Na Liang[1], Siyuan Wang[3], Pengbin Yin[4,5], Yuanping Yang[2], Sin Man Lam[6, 7], Qianying Zhang[1], Alimujiang Tudiyusufu[1], Yingying Gu[1], Xin Wan[1], Meihong Chen[1], Hu Li[2], Xiaofei Zhang[2], Guanghou Shui[6,7], Suneng Fu[8], Licheng Zhang[4,5], Peifu Tang[4,5], Catherine C.L. Wong[3], Yong Zhang[1,2*], Dahai Zhu[1,2*].

The supplementary information includes: Supplementary Figure 1–12 and the figure legends

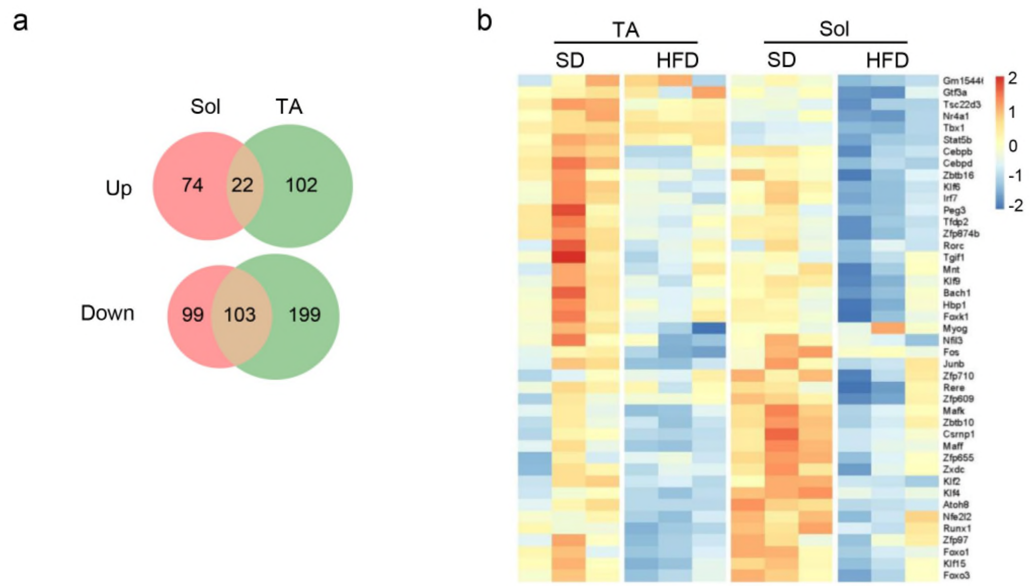

Supplementary Fig. 1 *Myod* is upregulated in TA muscle, but not in Sol muscle, in response to HFD feeding

a Venn diagram showing the numbers of upregulated (Up) and downregulated (Down) genes in TA and Sol muscles from HFD-fed mice compared to SD controls. **b** Heatmap showing the transcription factors (TFs) downregulated in TA and Sol muscles from HFD-fed mice compared to SD controls.

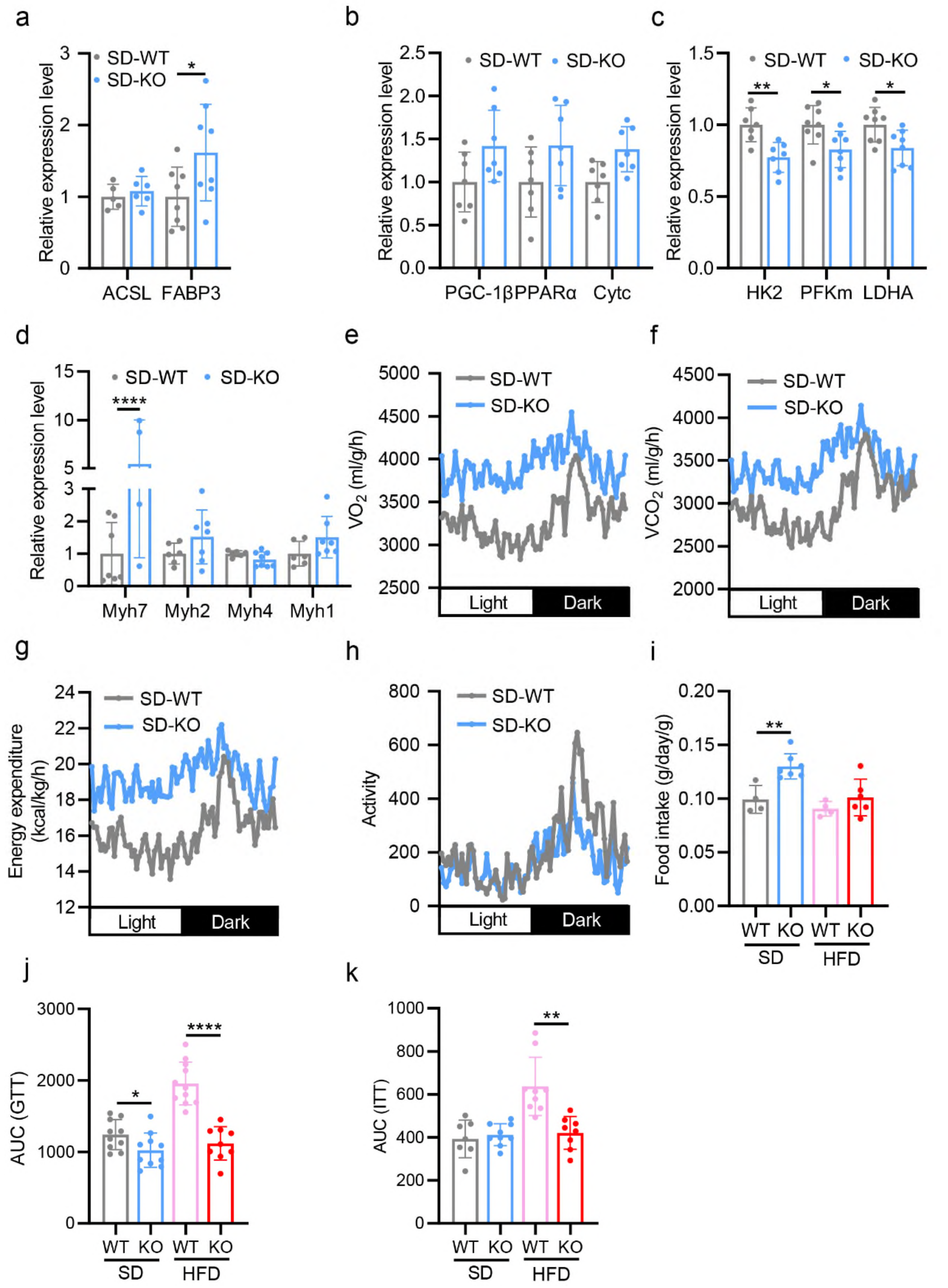

Supplementary Fig. 2 *Myod* KO mice exhibit enhanced oxidative metabolism in skeletal muscle and resist HFD-induced obesity

a Relative level of the mRNAs encoding long-chain acyl-CoA synthetase (*ACSL*) and fatty acid binding protein 3 (*FABP3*) in *quadriceps* (Qu) muscle of *Myod* KO mice and WT littermates fed with SD, determined by RT-qPCR. For *ACSL* gene, n = 5 (SD-WT), n = 6 (SD-KO); For

FABP3 gene, *n* = 8 (SD-WT), *n* = 8 (SD-KO) mice. *p* = 0.0242 (SD-WT vs. SD-KO). **b** Relative levels of the mRNAs encoding peroxisome proliferator-activated receptor gamma coactivator 1 beta (*PGC-1β*), peroxisome proliferator-activated receptor alpha (*PPARα*), and cytochrome C (*CytC*) in Qu muscle of *Myod* KO mice and WT littermates fed with SD, as determined by RT-qPCR. *n* = 7 mice. **c** Relative levels of genes encoding key enzymes for glycolysis in Qu muscle of *Myod* KO mice and WT littermates fed with SD, as determined by RT-qPCR. For *HK2* gene, *n* = 7 (SD-WT), *n* = 8 (SD-KO) mice. *p* = 0.0037 (SD-WT vs. SD-KO); For *PFKm* gene, *n* = 8 (SD-WT), *n* = 8 (SD-KO) mice. *p* = 0.0220 (SD-WT vs. SD-KO); For *LDHA* gene, *n* = 8 (SD-WT), *n* = 8 (SD-KO) mice. *p* = 0.0333 (SD-WT vs. SD-KO). *HK2*, hexokinase 2. *PFKm*, phosphofructokinase. *LDHA*, lactate dehydrogenase A. **d** Relative levels of genes encoding versions of fiber type-specific myosin-heavy chain (MHC), including *Myh7* (encoding MHC-I), *Myh2* (encoding MHC-IIa), *Myh1* (encoding MHC-IIx), and *Myh4* (encoding MHC-IIb), in Qu muscle of *Myod* KO mice and WT littermates fed with SD, as determined by RT-qPCR. For *Myh7* gene, *n* = 7 (SD-WT), *n* = 4 (SD-KO) mice. *p* < 0.0001 (SD-WT vs. SD-KO); For *Myh2* gene, *n* = 6 (SD-WT), *n* = 7 (SD-KO) mice; For *Myh4* gene, *n* = 6 (SD-WT), *n* = 8 (SD-KO) mice; For *Myh1* gene, *n* = 6 (SD-WT), *n* = 7 (SD-KO) mice. **e** O_2 consumption by *Myod* KO mice and WT littermates fed with SD, as determined by metabolic chamber analysis, *n* = 4 mice. **f** CO_2 production by *Myod* KO mice and WT littermates fed with SD, as determined by metabolic chamber analysis, *n* = 4 mice. **g** Energy expenditure by *Myod* KO mice and WT littermates fed with SD, as determined by metabolic chamber analysis, *n* = 4 mice. **h** Locomotor activities of *Myod* KO mice and WT littermates fed with SD, as determined by metabolic chamber analysis, *n* = 4 mice. **i** Food intake by *Myod* KO mice and WT littermates fed with HFD or SD, *n* = 4 (SD-WT), *n* = 7 (SD-KO), *n* = 4 (HFD-WT), *n* = 6 (HFD-KO) mice. *p* = 0.0041 (SD-WT vs. SD-KO). **j** Quantification of the area under the curve (AUC) from the GTT shown in Fig. 2h. *n* = 10 (SD-WT), *n* = 10 (SD-KO), *n* = 11 (HFD-WT), *n* = 9 (HFD-KO) mice. *p* = 0.0452 (SD-WT vs. SD-KO), *p* < 0.0001 (HFD-WT vs. HFD-KO). **k** Quantification of the area under the curve (AUC) from the ITT shown in Fig. 2i. *n* = 7 (SD-WT), *n* = 9 (SD-KO), *n* = 9 (HFD-WT), *n* = 8 (HFD-KO) mice. *p* = 0.0012 (HFD-WT vs. HFD-KO). Data are presented as mean ± SD. Significance was assessed by two-way ANOVA (a-d) or two tail Student's *t*-test (i-k). **p* < 0.05, ***p* < 0.01, *****p* < 0.0001 compared to WT control group.

Source data are provided as a Source Data file.

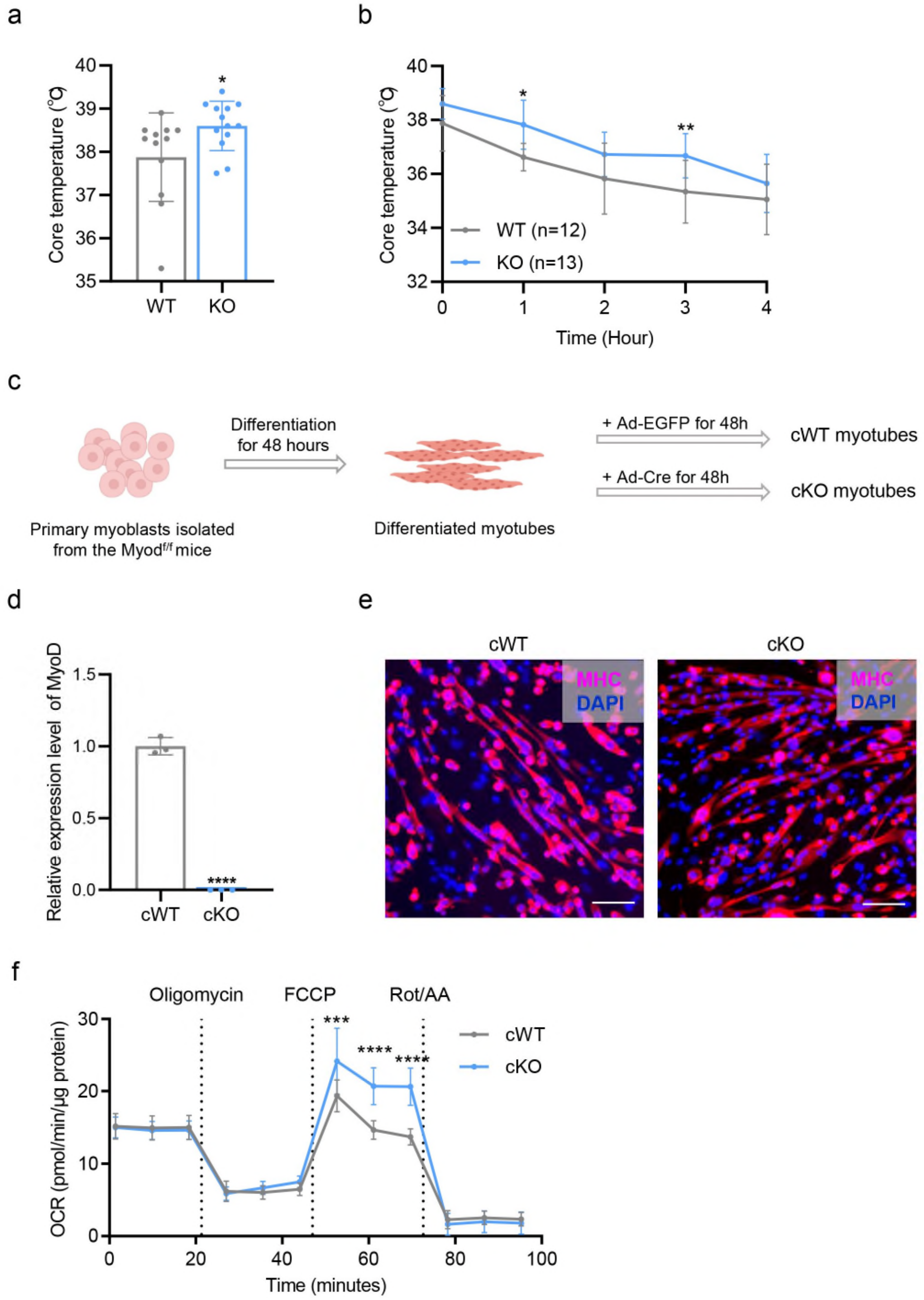

Supplementary Fig. 3 Knockout of *Myod* might enhance mitochondrial respiration.

a Core temperature in *Myod* KO mice (KO) and their WT controls at the thermal neutral conditions. n = 12 mice (WT), n = 13 mice (KO). p = 0.0373. **b** Core temperature in KO mice and their WT controls at 4°C for the indicated time points (0, 1, 2, 3, 4 h). n = 12 mice (WT), n = 13 mice (KO). n = 12 mice (WT), n = 13 mice (KO). p = 0.0145 (1 h), p = 0.0048 (3 h). **c**

Schematic diagram showing acute deletion of *Myod* in differentiated myotubes. The primary myoblasts isolated from the *Myod*$^{f/f}$ mice were induced to differentiation for 48 h and then infected with adenovirus expressing Cre (Ad-Cre) to achieve deletion of *Myod* (cKO), infection with the adenovirus expressing EGFP (Ad-EGFP) as controls (cWT). **d** Relative levels of *Myod* mRNA in cKO and cWT myotubes, as determined by RT-qPCR. $p < 0.0001$. Data are representative of three independent experiments. **e** Representative images showing immunostaining of myosin heavy chain (MHC) (red), a marker for the differentiated myotube, in cKO and cWT myotubes. DAPI (blue) served to visualize nuclei. Scale bar, 100 µm. **f** Oxygen consumption rate (OCR), determined by Seahorse XFe24; $p = 0.0007$ (52.63 min), $p < 0.0001$ (61.13 min), p < 0.0001 (69.63 min). Data are representative of three independent experiments. Data are presented as mean ± SD. Significance was assessed by two-way ANOVA (b, f) or two tail Student's *t*-test (a, d). *$p < 0.05$, **$p < 0.01$, ***$p < 0.001$, ****$p < 0.0001$ compared to WT or cWT control group. Source data are provided as a Source Data file.

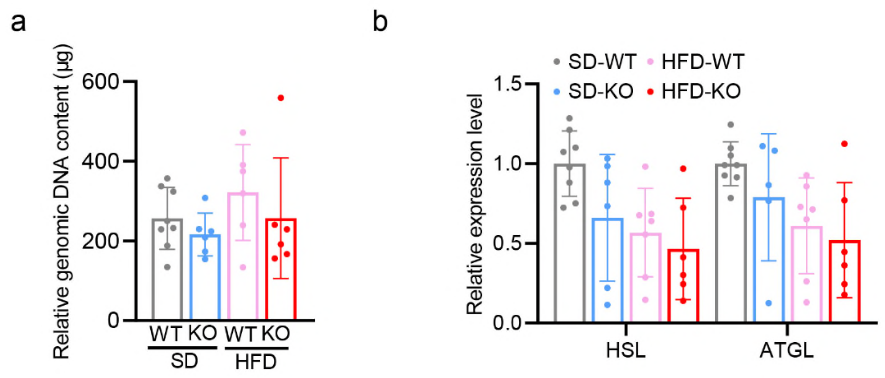

Supplementary Fig. 4 iWAT browning occurs in *Myod* KO mice

a Measurement of DNA contents in individual fat pads of iWAT from *Myod* KO mice and WT littermates fed with SD or HFD, *n* = 8 (SD-WT), *n* = 6 (SD-KO), *n* = 6 (HFD-WT), *n* = 6 (HFD-KO) mice. **b** Relative mRNA levels of lipolysis-related genes in iWAT from *Myod* KO mice and WT littermates fed with SD or HFD, as determined by RT-qPCR. For *HSL* gene, *n* = 8 (SD-WT), *n* = 6 (SD-KO), *n* = 7 (HFD-WT), *n* = 6 (HFD-KO) mice; For *ATGL* gene, *n* = 8 (SD-WT), *n* = 5 (SD-KO), *n* = 7 (HFD-WT), *n* = 6 (HFD-KO) mice. *HSL*, hormone-sensitive lipase. *ATGL*, adipose triglyceride lipase. Data are presented as mean ± SD. Significance was assessed by two-way ANOVA (b) or two tail Student's *t*-test (a). Source data are provided as a Source Data file.

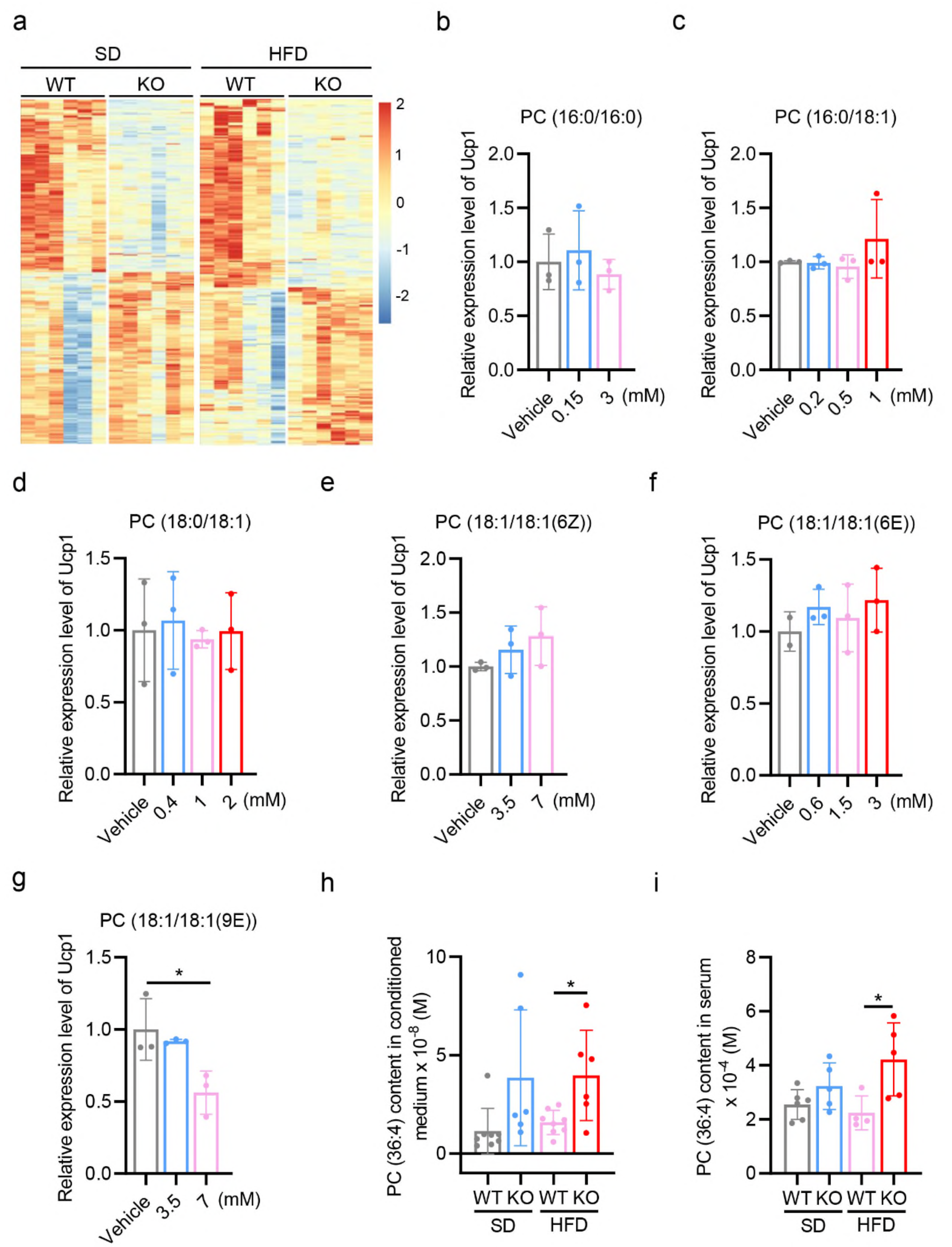

Supplementary Fig. 5 DLPC induces iWAT browning in HFD-fed *Myod* KO mice

a Heatmap showing DEGs in TA and Sol muscles of *Myod* KO mice versus WT littermates fed with SD or HFD for 2 weeks, as described in Fig. 4a. **b-g** Relative mRNA levels of *Ucp1* in iWAT-derived primary adipocytes treated with vehicle or various doses of the indicated PC species for 12 h, as determined by RT-qPCR. $p < 0.0210$ (Vehicle vs. 7mM PC (18:1/18:1(9E))). Data are representative of three independent experiments. **h** Levels of PC (36:4) in muscle-

derived CM from the mice described in (Fig. 4a); n = 8 (SD-WT), n= 6 (SD-KO), n= 8 (HFD-WT), n= 6 (HFD-KO) mice. $p = 0.0148$ (HFD-WT vs. HFD-KO). **i** Levels of PC (36:4) in sera collected from the mice described in (Fig. 4a); n = 6 (SD-WT), n= 5 (SD-KO), n= 4 (HFD-WT), n= 5 (HFD-KO) mice. $p = 0.0314$ (HFD-WT vs. HFD-KO). Data are presented as mean ± SD. Significance was assessed by one-way ANOVA (b-g) or two tail Student's *t*-test (h-i). *$p < 0.05$ compared to vehicle control group. Source data are provided as a Source Data file.

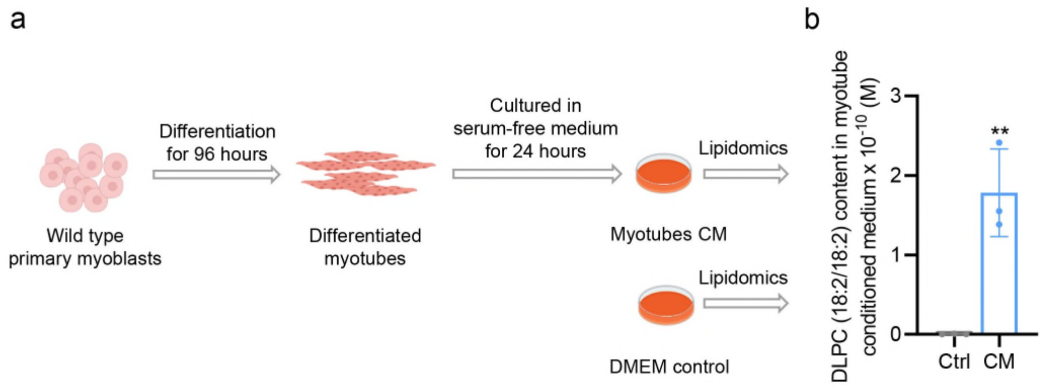

Supplementary Fig. 6 DLPC is detected in the conditioned medium from the differentiated myotubes.

a Experimental scheme for using lipidomics analyses to detect myotubes-secreted DLPC from conditioned medium (CM). b Levels of DLPC (18:2/ 18:2) in myotubes-derived CM. p = 0.0050. Data are representative of three independent experiments. Data are presented as mean ± SD. Significance was assessed by two tail Student's *t*-test (b). $^{**}p < 0.01$, compared to DMEM (Ctrl) control. Source data are provided as a Source Data file.

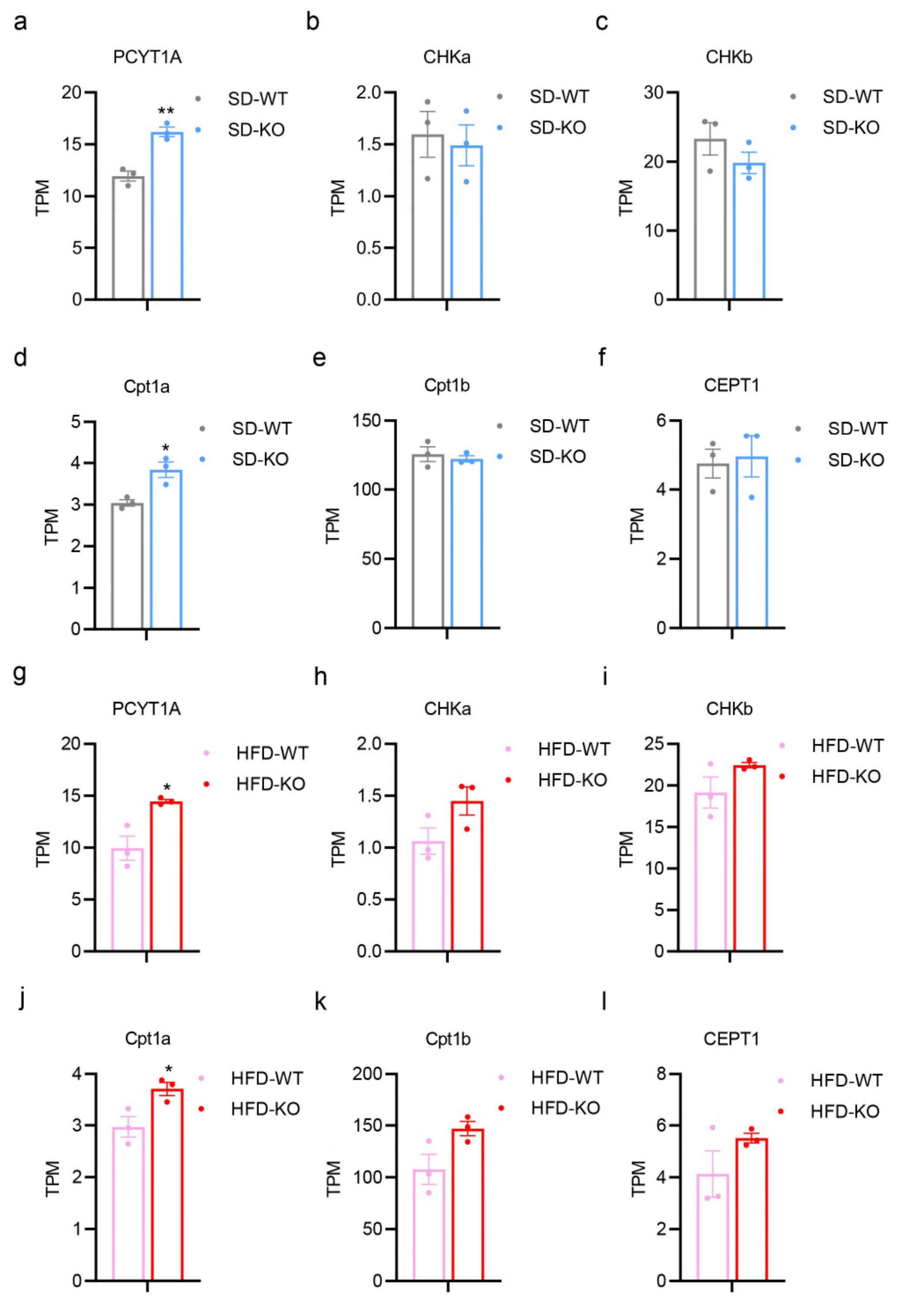

Supplementary Fig. 7 Expression levels of the genes involved in the PC biosynthesis pathway in Soleus muscle of *Myod* KO mice

a-l The data (TPM) were determined by RNA-seq. n = 3 mice. p = 0.0030 (*PCYT1A*, SD-WT vs. SD-KO), p = 0.0170 (*Cpt1a*, SD-WT vs. SD-KO), *p* = 0.0186 (HFD-WT vs. HFD-KO), *p*

= 0.0356 (HFD-WT vs. HFD-KO). Data are presented as mean ± SD. Significance was assessed by two tail Student's *t*-test (a-l). *$p < 0.05$, **$p < 0.01$, compared to WT. Source data are provided as a Source Data file.

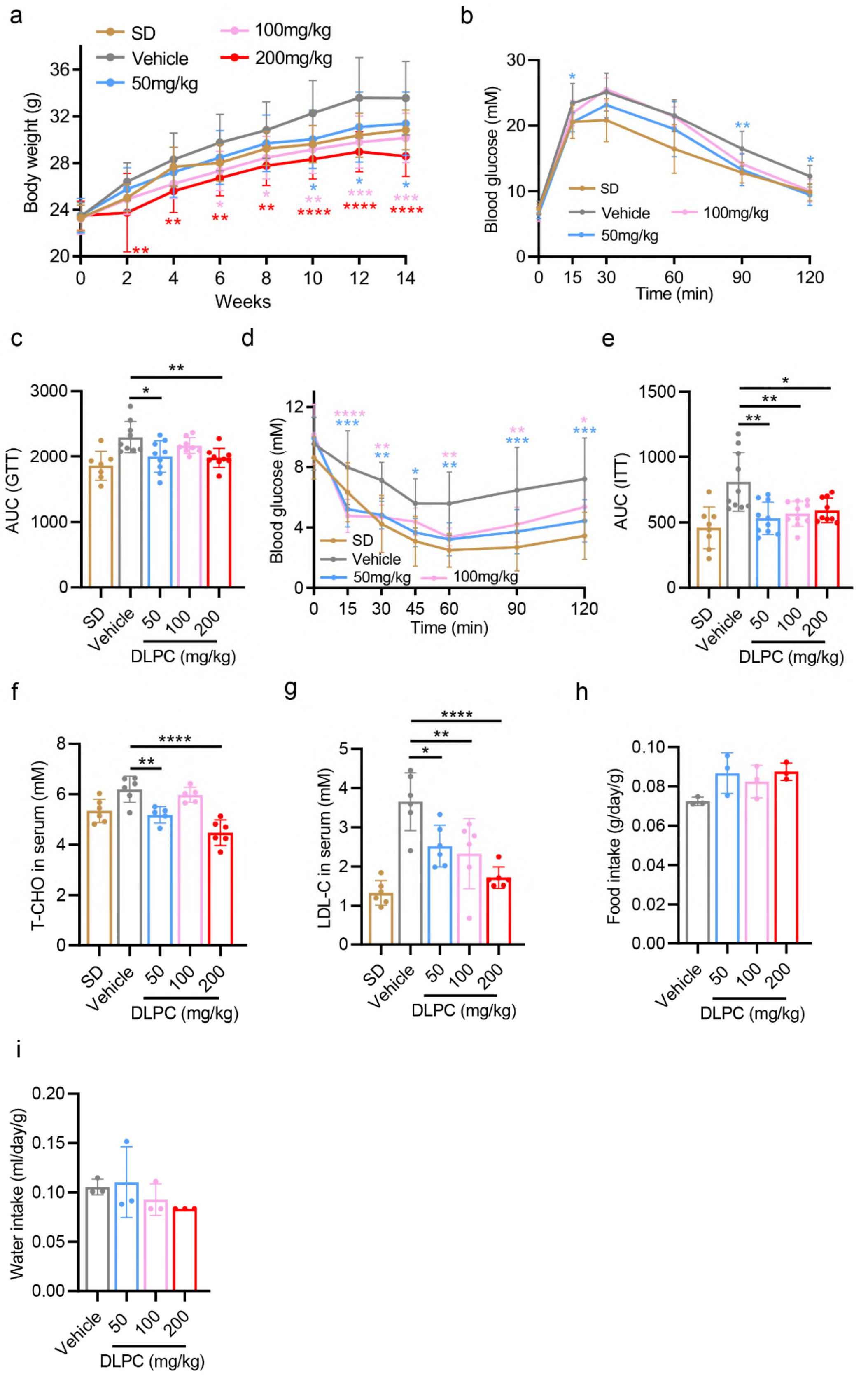

Supplementary Fig. 8 DLPC prevents HFD-induced obesity

a Body weights of mice that were fed with SD or HFD and simultaneously i.p. administered with vehicle or various doses of DLPC (50, 100, 200 mg/kg) daily for 14 weeks, n = 8 (SD), n = 11 (Vehicle), n = 11 (50 mg/kg), n = 11 (100 mg/kg), n = 12 (200 mg/kg) mice. For Vehicle vs. 50mg/kg, p = 0.0437 (10-week), p = 0.0186 (12-week), p = 0.0499 (14-week); For Vehicle vs. 100mg/kg, p = 0.0250 (6-week), p = 0.0280 (8-week), p = 0.0020 (10-week), p = 0.0001 (12-week), p = 0.0006 (14-week); For Vehicle vs. 200mg/kg, p = 0.0091 (2-week), p = 0.0076 (4-week), p = 0.0021 (6-week), p = 0.0019 (8-week), $p < 0.0001$ (10-week), $p < 0.0001$ (12-week), $p < 0.0001$ (14-week). **b** GTT performance of the mice described in (Fig. 5a); n = 7 (SD), n = 9 (Vehicle), n = 9 (50 mg/kg), n = 8 (100 mg/kg) mice. For Vehicle vs. 50mg/kg, p = 0.0185 (15 min), p = 0.0072 (90 min), p = 0.0202 (120 min). **c** Quantification of the area under the curve (AUC) from the GTT shown in Fig. 5h and Supplementary Fig. 5b. n = 7 (SD), n = 9 (Vehicle), n = 9 (50 mg/kg), n = 8 (100 mg/kg), n = 9 (200 mg/kg) mice. p = 0.0199 (Vehicle vs. 50mg/kg), p = 0.0040 (Vehicle vs. 200mg/kg). **d** ITT performance of the mice described in Fig. 5a. n = 8 (SD), n = 10 (Vehicle), n = 10 (50 mg/kg), n = 10 (100 mg/kg) mice. For Vehicle vs. 50mg/kg, p = 0.0005 (15 min), p = 0.0049 (30 min), p = 0.0230 (45 min), p = 0.0038 (60 min), p = 0.0006 (90 min), p = 0.0005 (120 min); For Vehicle vs. 100mg/kg, $p < 0.0001$ (15 min), p = 0.0027 (30 min), p = 0.0007 (60 min), p = 0.0059 (90 min), p = 0.0311 (120 min). **e** Quantification of the area under the curve (AUC) from the ITT shown in Fig. 5i and Supplementary Fig. 5d. n = 8 (SD), n = 10 (Vehicle), n = 10 (50 mg/kg), n = 10 (100 mg/kg), n = 9 (200 mg/kg) mice. p = 0.0029 (Vehicle vs. 50mg/kg), p = 0.0054 (Vehicle vs. 100mg/kg), p = 0.0152 (Vehicle vs. 200mg/kg). **f** Total cholesterol (T-CHO) in sera from the mice described in (Fig. 5a); n = 6 (SD), n = 6 (Vehicle), n = 5 (50 mg/kg), n = 5 (100 mg/kg), n = 6 (200 mg/kg) mice. p = 0.0038 (Vehicle vs. 50mg/kg), $p < 0.0001$ (Vehicle vs. 200mg/kg). **g** Low density lipoprotein cholesterol (LDL-C) in sera of the mice described in (Fig. 5a); n = 6 mice. p = 0.0109 (Vehicle vs. 50mg/kg), p = 0.0028 (Vehicle vs. 100mg/kg), $p < 0.0001$ (Vehicle vs. 200mg/kg). **h** Food intake by the mice described in (Fig. 5a), n = 3 biologically independent experiments. **i** Water intake by the mice described in (Fig. 5a), n = 3 biologically independent experiments. Data are presented as mean ± SD. Significance was assessed by one-way ANOVA (f-i), two-way ANOVA (a-b, d), or two tail Student's *t*-test (c, e). $^{*}p < 0.05$, $^{**}p < 0.01$, $^{***}p < 0.001$, $^{****}p < 0.0001$ compared to vehicle control group. Source data are provided as a Source

Data file.

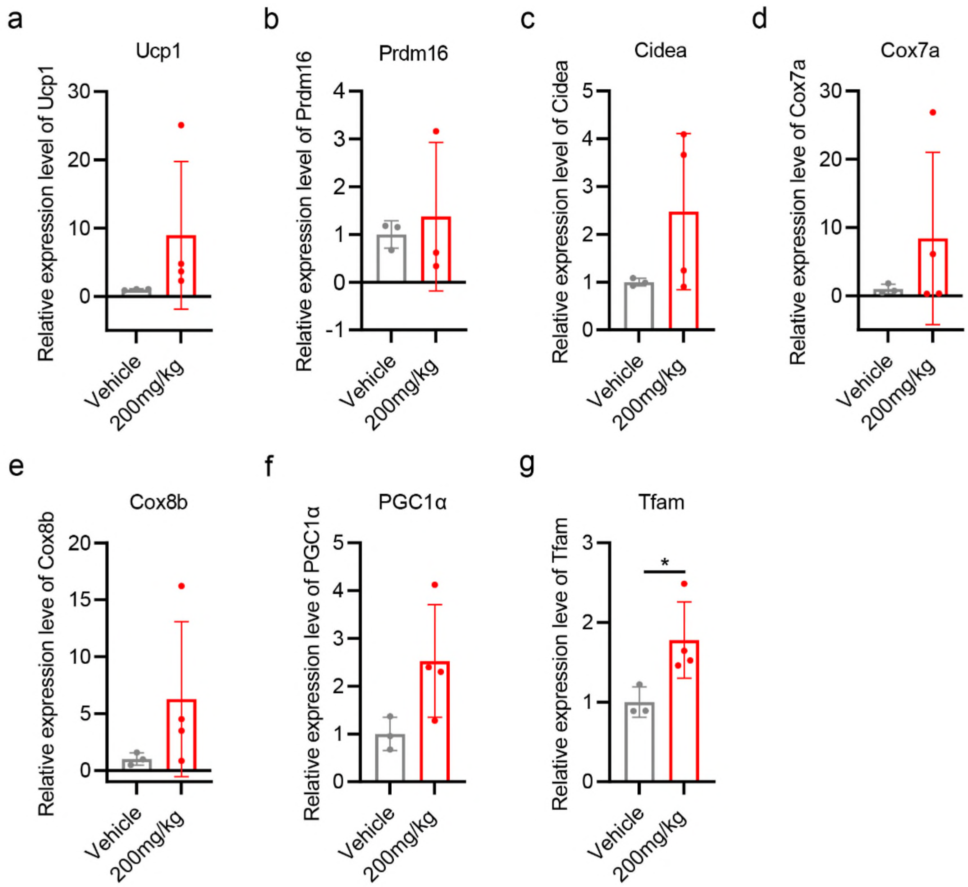

Supplementary Fig. 9 DLPC prevents HFD-induced obesity

a-g Relative mRNA levels of browning-related genes in iWAT of the mice described in Fig. 5a, as determined by RT-qPCR. For *Ucp1* gene, *n* = 3 (Vehicle), *n* = 4 (200 mg/kg) mice; For *Prdm16* gene, *n* = 3 (Vehicle), *n* = 3 (200 mg/kg) mice; For *Cidea* gene, *n* = 3 (Vehicle), *n* = 4 (200 mg/kg) mice; For *Cox7a* gene, *n* = 3 (Vehicle), *n* = 4 (200 mg/kg) mice; For *Cox8b* gene, *n* = 3 (Vehicle), *n* = 4 (200 mg/kg) mice; For *PGC1α* gene, *n* = 3 (Vehicle), *n* = 4 (200 mg/kg) mice; For *Tfam* gene, *n* = 3 (Vehicle), *n* = 4 (200 mg/kg) mice. *p* = 0.0471 (Vehicle vs. 200mg/kg). Data are presented as mean ± SD. Significance was assessed by two tail Student's *t*-test (a-g). $^*p < 0.05$ compared to vehicle control group. Source data are provided as a Source Data file.

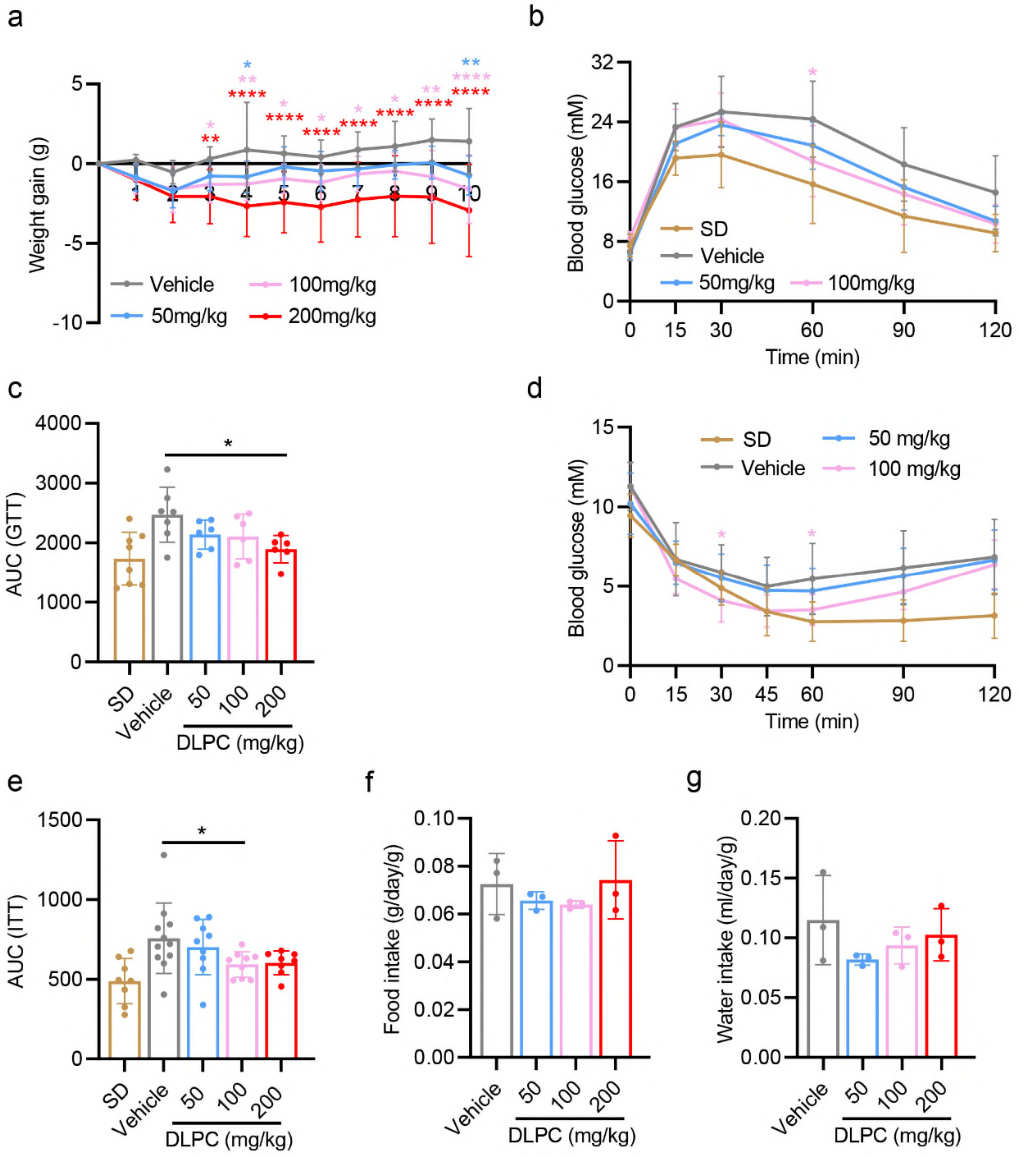

Supplementary Fig. 10 DLPC treats obesity in DIO mice

a Weight gain of DIO mice that were i.p. administered with vehicle or various doses of DLPC (50, 100, 200 mg/kg) daily for 10 weeks. n = 10 (Vehicle), n = 10 (50 mg/kg), n = 12 (100 mg/kg), n = 10 (200 mg/kg) mice. For Vehicle vs. 50mg/kg, p = 0.0313 (4-week), p = 0.0039 (10-week); For Vehicle vs. 100mg/kg, p = 0.0314 (3-week), p = 0.0023 (4-week), p = 0.0342 (5-week), p = 0.0337 (6-week), p = 0.0456 (7-week), p = 0.0380 (8-week), p = 0.0010 (9-week), $p < 0.0001$ (10-week); For Vehicle vs. 200mg/kg, p = 0.0011 (3-week), $p < 0.0001$ (4-week), $p < 0.0001$ (5-week), $p < 0.0001$ (6-week), $p < 0.0001$ (7-week), $p < 0.0001$ (8-week), $p < 0.0001$ (9-week), $p < 0.0001$ (10-week). Mice treated with vehicle served as controls. **b** GTT performance of the mice described in Fig. 6a. n = 8 (SD), n = 7 (Vehicle), n = 6 (50 mg/kg), n

= 6 (100 mg/kg) mice. For Vehicle vs. 100mg/kg, $p = 0.0134$ (60 min). **c** Quantification of the area under the curve (AUC) from the GTT shown in Fig. 6d and Supplementary Fig. 7b. $n = 8$ (SD), $n = 7$ (Vehicle), $n = 6$ (50 mg/kg), $n = 6$ (100 mg/kg), $n = 6$ (200 mg/kg) mice. $p = 0.0182$ (Vehicle vs. 200mg/kg) **d** ITT performance of the mice described in Fig. 6a. $n = 8$ (SD), $n = 11$ (Vehicle), $n = 9$ (50 mg/kg), $n = 9$ (100 mg/kg) mice. For Vehicle vs. 100mg/kg, $p = 0.0461$ (30 min), $p = 0.0241$ (60 min). **e** Quantification of the area under the curve (AUC) from the ITT shown in Fig. 6e and Supplementary Fig. 7d. $n = 8$ (SD), $n = 11$ (Vehicle), $n = 9$ (50 mg/kg), $n = 9$ (100 mg/kg), $n = 8$ (200 mg/kg) mice. $p = 0.0480$ (Vehicle vs. 100mg/kg). **f** Food intake by the mice described in Fig. 6a, $n = 3$ biologically independent experiments. **g** Water intake by the mice described in Fig. 6a, $n = 3$ biologically independent experiments. Data are presented as mean ± SD. Significance was assessed by one-way ANOVA (f-g), two-way ANOVA (a, b, d), or two tail Student's *t*-test (c, e). $^{*}p < 0.05$, $^{**}p < 0.01$, $^{***}p < 0.001$, $^{****}p < 0.0001$ compared to vehicle control group. Source data are provided as a Source Data file.

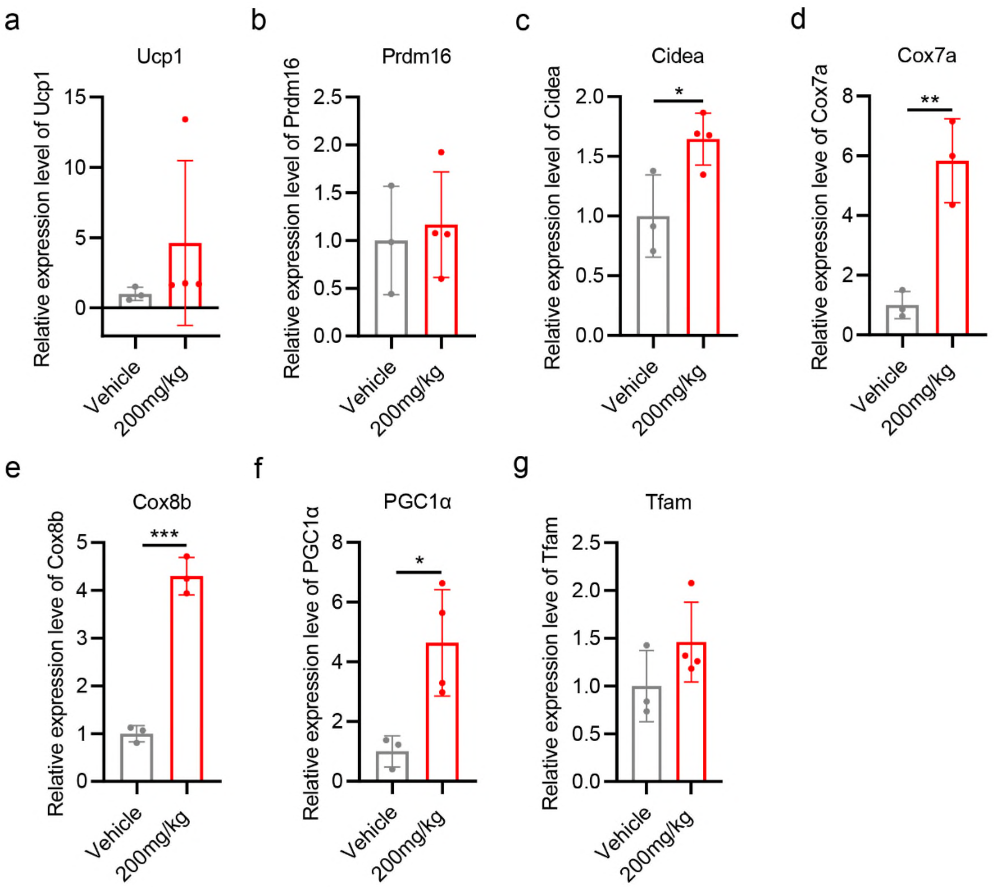

Supplementary Fig. 11 DLPC treats obesity in DIO mice

a-g Relative mRNA levels of browning-related genes in iWAT tissues of the mice described in (Fig. 6a), as determined by RT-qPCR. For *Ucp1* gene, $n = 3$ (Vehicle), $n = 4$ (200 mg/kg) mice; For *Prdm16* gene, $n = 3$ (Vehicle), $n = 4$ (200 mg/kg) mice; For *Cidea* gene, $n = 3$ (Vehicle), $n = 4$ (200 mg/kg) mice, $p = 0.0277$ (Vehicle vs. 200mg/kg); For *Cox7a* gene, $n = 3$ (Vehicle), $n = 3$ (200 mg/kg) mice, $p = 0.0048$ (Vehicle vs. 200mg/kg); For *Cox8b* gene, $n = 3$ (Vehicle), $n = 3$ (200 mg/kg) mice, $p = 0.0002$ (Vehicle vs. 200mg/kg); For *PGC1α* gene, $n = 3$ (Vehicle), $n = 4$ (200 mg/kg) mice, $p = 0.0204$ (Vehicle vs. 200mg/kg); For *Tfam* gene, $n = 3$ (Vehicle), $n = 4$ (200 mg/kg) mice. Data are presented as mean ± SD. Significance was assessed by two tail Student's *t*-test (a-g). $^{*}p < 0.05$, $^{**}p < 0.01$, $^{***}p < 0.001$ compared to vehicle control group. Source data are provided as a Source Data file.

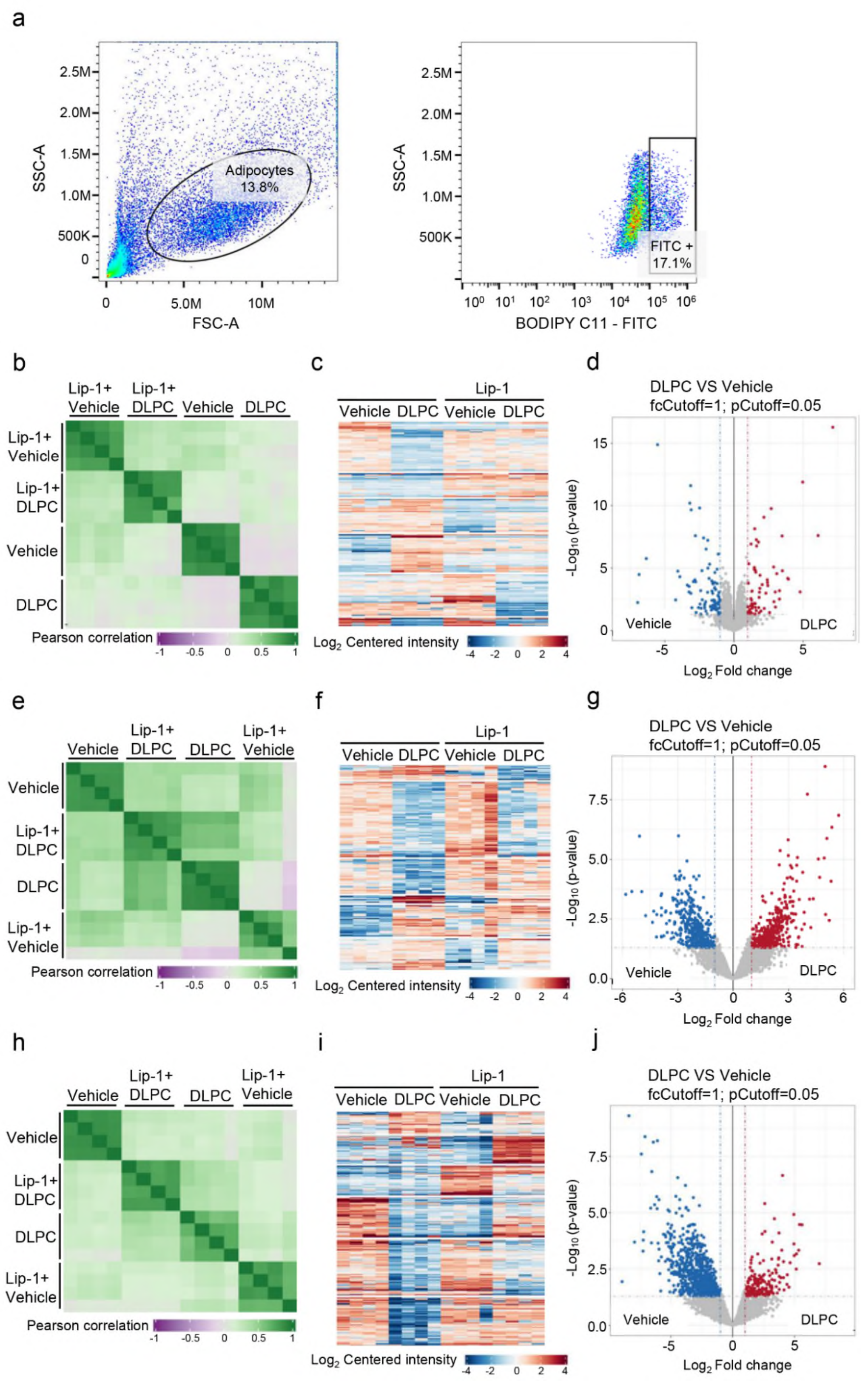

Supplementary Fig. 12 DLPC induces iWAT browning via lipid peroxidation-mediated p38 activation

a Gating strategy used for FACS sorting of BODIPY C11 positive cells. **b** Pearson correlation matrix of quantitative proteomics data. **c** Heatmap showing proteins that were differentially expressed between DLPC-treated and vehicle control adipocytes in the absence of Lip-1, as determined by proteomics analysis. **d** Volcano plot showing proteins that were differentially expressed between DLPC-treated and vehicle control adipocytes in the absence of Lip-1, as determined by proteomics analysis. **e** Pearson correlation matrix of quantitative redox proteomics data. **f** Heatmap showing proteins that were differentially expressed between DLPC-treated and vehicle control adipocytes in the absence of Lip-1, as determined by redox proteomics analysis. **g** Volcano plot showing the differentially oxidized proteins between DLPC-treated and vehicle control adipocytes in the absence of Lip-1, as determined by redox proteomics analysis. **h** Pearson correlation matrix of quantitative phospho-proteomics data. **i** Heatmap showing proteins that were differentially expressed between DLPC-treated and vehicle control adipocytes in the absence of Lip-1, as determined by phospho-proteomics analysis. **j** Volcano plot showing phospho-proteins that were differentially present between DLPC-treated and vehicle control adipocytes in the absence of Lip-1, as determined by phospho-proteomics analysis.
