# Supplementary material for: Skeletal muscle-secreted DLPC orchestrates systemic energy homeostasis by enhancing adipose browning
Source: Nat Commun. 2023 Nov 30;14:7916. doi: 10.1038/s41467-023-43402-z (PMC10689447; doi:10.1038/s41467-023-43402-z)
Supplement: Supplementary file 3 — Description of Additional Supplementary Files [file 41467_2023_43402_MOESM3_ESM.pdf]

Description of Additional Supplementary Files

Supplementary Datasets

Supplementary Data 1. Conditional medium metabolomics

Supplementary Data 2. Serum metabolomics

Supplementary Data 3. Conditional medium lipidomics

Supplementary Data 4. Serum lipidomics
