# Supplementary material for: Skeletal muscle-secreted DLPC orchestrates systemic energy homeostasis by enhancing adipose browning
Source: Nat Commun. 2023 Nov 30;14:7916. doi: 10.1038/s41467-023-43402-z (PMC10689447; doi:10.1038/s41467-023-43402-z)
Supplement: Supplementary file 8 — Reporting Summary [file 41467_2023_43402_MOESM8_ESM.pdf]

nature portfolio

Corresponding author(s): Dahai Zhu

Last updated by author(s): Oct 27, 2023

Reporting Summary

Nature Portfolio wishes to improve the reproducibility of the work that we publish. This form provides structure for consistency and transparency in reporting. For further information on Nature Portfolio policies, see our Editorial Policies and the Editorial Policy Checklist.

Statistics

For all statistical analyses, confirm that the following items are present in the figure legend, table legend, main text, or Methods section.

n/a	Confirmed	
☐	☒	The exact sample size (*n*) for each experimental group/condition, given as a discrete number and unit of measurement
☐	☒	A statement on whether measurements were taken from distinct samples or whether the same sample was measured repeatedly
☐	☒	The statistical test(s) used AND whether they are one- or two-sided<br>*Only common tests should be described solely by name; describe more complex techniques in the Methods section.*
☒	☐	A description of all covariates tested
☐	☒	A description of any assumptions or corrections, such as tests of normality and adjustment for multiple comparisons
☐	☒	A full description of the statistical parameters including central tendency (e.g. means) or other basic estimates (e.g. regression coefficient) AND variation (e.g. standard deviation) or associated estimates of uncertainty (e.g. confidence intervals)
☐	☒	For null hypothesis testing, the test statistic (e.g. *F*, *t*, *r*) with confidence intervals, effect sizes, degrees of freedom and *P* value noted<br>*Give P values as exact values whenever suitable.*
☒	☐	For Bayesian analysis, information on the choice of priors and Markov chain Monte Carlo settings
☒	☐	For hierarchical and complex designs, identification of the appropriate level for tests and full reporting of outcomes
☒	☐	Estimates of effect sizes (e.g. Cohen's *d*, Pearson's *r*), indicating how they were calculated

Our web collection on statistics for biologists contains articles on many of the points above.

Software and code

Policy information about availability of computer code

Data collection	No special software was used.
Data analysis	DESeq2 (Version 1.24.0) BD Accuri C6 Plus Software (Version 1.0.23.1) FlowJo (Version 10.8.1) GraphPad Prism (Version 9.4.1), ClusterProfiler (Version 3.14), WAVE software (Version 2.6.1.53)

For manuscripts utilizing custom algorithms or software that are central to the research but not yet described in published literature, software must be made available to editors and reviewers. We strongly encourage code deposition in a community repository (e.g. GitHub). See the Nature Portfolio guidelines for submitting code & software for further information.

Data

Policy information about availability of data

All manuscripts must include a data availability statement. This statement should provide the following information, where applicable:

- Accession codes, unique identifiers, or web links for publicly available datasets
- A description of any restrictions on data availability
- For clinical datasets or third party data, please ensure that the statement adheres to our policy

The raw sequence data reported in this paper have been deposited in the Genome Sequence Archive in National Genomics Data Center, China National Center for Bioinformation / Beijing Institute of Genomics, Chinese Academy of Sciences (GSA: CRA010866) that are publicly accessible at https://ngdc.cncb.ac.cn/gsa.

Research involving human participants, their data, or biological material

Policy information about studies with human participants or human data. See also policy information about sex, gender (identity/presentation), and sexual orientation and race, ethnicity and racism.

Reporting on sex and gender	Not applicable.
Reporting on race, ethnicity, or other socially relevant groupings	Not applicable.
Population characteristics	Not applicable.
Recruitment	Not applicable.
Ethics oversight	Not applicable.

Note that full information on the approval of the study protocol must also be provided in the manuscript.

Field-specific reporting

Please select the one below that is the best fit for your research. If you are not sure, read the appropriate sections before making your selection.

☒ Life sciences ☐ Behavioural & social sciences ☐ Ecological, evolutionary & environmental sciences

For a reference copy of the document with all sections, see nature.com/documents/nr-reporting-summary-flat.pdf

Life sciences study design

All studies must disclose on these points even when the disclosure is negative.

Sample size	Sample size was not determined by specific statistical methods, but were based on variability of associated assays. Three biological replicates for RNA-seq and 8-12 biological replicates for the phenotype analysis were used in the study.
Data exclusions	All data were included, without specific exclusions.
Replication	All experiments were reproducible. Every figure states how many times the related experiments was performed with similar results. All data presented were from independent biological replicates or independent experiments.
Randomization	For phenotype observation, animals were randomly assigned to each individual groups.
Blinding	For phenotype observation, the data analysis was performed blind to genotype and treatment.

Reporting for specific materials, systems and methods

We require information from authors about some types of materials, experimental systems and methods used in many studies. Here, indicate whether each material, system or method listed is relevant to your study. If you are not sure if a list item applies to your research, read the appropriate section before selecting a response.

Materials & experimental systems

n/a	Involved in the study	
☐	☒	Antibodies
☒	☐	Eukaryotic cell lines
☒	☐	Palaeontology and archaeology
☐	☒	Animals and other organisms
☒	☐	Clinical data
☒	☐	Dual use research of concern
☒	☐	Plants

Methods

n/a	Involved in the study	
☒	☐	ChIP-seq
☐	☒	Flow cytometry
☒	☐	MRI-based neuroimaging

Antibodies

Antibodies used	Ucp antibody, Rabbit polyclonal (Abcam, ab10983; lot. no. GR3432957-1; dilution, 1:300 for WB);<br>Phospho-p38 MAPK (Thr180/Tyr182) antibody, Rabbit polyclonal (Cell Signaling Technology, 9211s; lot. no.25; dilution, 1:1000 for WB);

p38 MAPK Antibody, Rabbit polyclonal (Cell Signaling Technology, 9212s; lot. no.26; dilution, 1:1000 for WB);
Anti-beta Tubulin Antibody, Mouse monoclonal (Engibody Biotechnology, AT0003; lot. no.422022BR-A0438; dilution, 1:4000 for WB)
MHC Antibody, Mouse monoclonal DSHB, MF20, lot. no. AB2147781; dilution, 1:500 for immunofluorescent staining)

Validation

All antibodies are purchased from commercial resources. Validation statements can be found on the manufacturer's website for the following:
Ucp1 antibody (Abcam, ab10983): https://www.abcam.cn/products/primary-antibodies/ucp1-antibody-ab10983.html
Phospho-p38 MAPK (Thr180/Tyr182) antibody (Cell Signaling Technology, 9211s): https://www.cellsignal.cn/products/primary-antibodies/phospho-p38-mapk-thr180-tyr182-antibody/9211?site-search-type=Products&N=4294956287&Ntt=9211s&fromPage=plp&_requestid=1710457
p38 MAPK antibody (Cell Signaling Technology, 9212s): https://www.cellsignal.cn/products/primary-antibodies/p38-mapk-antibody/9212?site-search-type=Products&N=4294956287&Ntt=9212s&fromPage=plp&_requestid=1711544
Anti-beta Tubulin antibody (Engibody Biotechnology, AT0003): https://www.engibody.com/products/beta-tubulin-mouse-mab-loading-control-at0003.html
MHC antibody (DSHB, MF20): https://dshb.biology.uiowa.edu/MF-20

Animals and other research organisms

Policy information about studies involving animals; ARRIVE guidelines recommended for reporting animal research, and Sex and Gender in Research

Laboratory animals

Mice were housed in a pathogen-free facility and had free access to water and standard rodent chow under the following conditions: 21°C ambient temperature, 50–60% humidity, 12 h dark/light cycle. Myod-knockout (KO) mice (#002523) were obtained from the Jackson Laboratory. C57BL/6j male mice were purchased from Beijing HFK Bioscience Co., Ltd. For studies with specific diets, 6-week-old male mice were fed with high-fat diet (HFD, 45% fat, Medicine Diets, MD12032) or standard diet (SD, 10% fat, Medicine Diets, MD12031) for a total period of 8 weeks or 12-16 weeks.

Wild animals

No wild animals were used in the study.

Reporting on sex

Male mice fed with high-fat diet or standard diet were used for the phenotype observation.

Field-collected samples

The study did not involve samples collected from the field.

Ethics oversight

All animal procedures were approved by the Animal Ethics Committee of Peking Union Medical College, Beijing (China).

Note that full information on the approval of the study protocol must also be provided in the manuscript.

Flow Cytometry

Plots

Confirm that:

☒ The axis labels state the marker and fluorochrome used (e.g. CD4-FITC).

☒ The axis scales are clearly visible. Include numbers along axes only for bottom left plot of group (a 'group' is an analysis of identical markers).

☒ All plots are contour plots with outliers or pseudocolor plots.

☒ A numerical value for number of cells or percentage (with statistics) is provided.

Methodology

Sample preparation

The differentiated adipocytes were treated with vehicle or 0.25 mM DLPC for 3 hours in the presence or absence of the lipid peroxidation inhibitor, liproxstatin-1 (Lip-1), and then stained with Bodipy C11. The cells were trypsinized and filtered through 70-µm and 40-µm cell strainers, suspended at 10^3–10^7 cells/ml.

Instrument

FACS was performed using Accuri C6 plus (BD Bioscience, America).

Software

Data were analyzed using BD Accuri C6 Plus Software (BD Bioscience, America), FlowJo (Version 10.8.1) (Becton Dickinson & Company (BD), America).

Cell population abundance

For each sample, at least 10000 cells were collected.

Gating strategy

FSC-A versus SSC-A was initially applied to the gate for adipocytes (main targets). The gate was set to remove cell debris (small FSC v SSC) and large clumps or aggregates of cells (large FSC or SSC), and used across all samples. Adipocytes were gated on single cells using SSC-A verus Bodipy C11 and then gated as BODIPY C11- FITC positive cells were targeted cells.

☒ Tick this box to confirm that a figure exemplifying the gating strategy is provided in the Supplementary Information.
